# The international HTA database returned incomplete search results for NICE technology appraisals: An exploratory study and discussion of the implications

**DOI:** 10.1002/cesm.12034

**Published:** 2023-12-01

**Authors:** Christopher Cooper, Sabrina Smith

**Affiliations:** ^1^ Population Health Sciences Bristol Medical School Bristol UK; ^2^ Stratenym Toronto ON Canada

**Keywords:** Health Technology Assessment, HTA, International Network of Agencies for Health Technology Assessment database, INAHTA, National Institute for Health and Care Excellence

## Abstract

**Introduction:**

The International Network of Agencies for Health Technology Assessment (INAHTA) database offers a single point of access for identifying technology appraisals, in contrast to searching multiple websites directly. The aim of this research is to compare the coverage of the INAHTA and Centre for Reviews and Dissemination (CRD) Health Technology Assessment (HTA) databases with direct searching on the National Institute for Health and Care Excellence (NICE) website to identify Technology Appraisals published by NICE.

**Methods:**

NICE Technology Appraisals were downloaded from the NICE website (April 2022). Technology Appraisals were randomized and the first 20 Technology Appraisals constituted data for analysis. The INAHTA and CRD HTA databases were searched to determine if the 20 Technology Appraisals available on the NICE website were also available for retrieval.

**Results:**

Coverage was incomplete. INAHTA: 15 of 20 Technology Appraisals (75%) were not identified via full title or intervention‐specific searches. CRD HTA: 7 of 12 Technology Appraisals (58%) that were published before the last update of the database were not identified.

**Conclusion:**

Findings indicate that researchers seeking to identify NICE Technology Appraisals should search the NICE website directly. How this finding impacts identification of guidance from other agencies should be evaluated.

## 1 INTRODUCTION

Systematic reviews supporting Health Technology Assessments (HTAs) commonly include searches for guidance. This guidance takes the form of Technology Appraisals, which report evaluations of products, devices, or technologies such as medicines [1], and which can be used to inform and to support submissions for reimbursement and systematic reviews. Technology Appraisals are published by guidance‐producing agencies, such as the National Institute for Health and Care Excellence (NICE) in the United Kingdom. [1]

Searches for Technology Appraisals can be undertaken directly, by searching the websites of guidance‐producing agencies, or indirectly through a search of either of the following federated and web‐based databases:

1. Centre for Reviews and Dissemination (CRD) Health Technology Assessment (HTA) database (https://www.crd.york.ac.uk/CRDWeb/). The CRD HTA database has not been updated since March 31, 2018, but it will be maintained until “at least March 2024” [2]; and
2. International Network of Agencies for Health Technology Assessment (INAHTA) database (https://database.inahta.org/). [3]

Both databases aim (or aimed, in the case of CRD HTA) to provide free access to completed and ongoing HTAs issued by HTA organizations worldwide. The INAHTA database claims that it offers “a single point of access to information that would otherwise be more difficult and time‐consuming to search for on individual agency websites.” [3] Core background data on each database is summarized in Table 1. As searching the websites of guidance‐producing agencies directly is labor‐intensive, there is substantial benefit to the research community in having a single point of access for HTAs, but only if the resource can be relied upon to be up to date and accurate.

**Table 1:** Brief description of INAHTA and CRD HTA databases.

	INAHTA database	CRD HTA database
Background	The INAHTA database was released in its current form in June 2020, with the aim to replace the CRD HTA database and provide a single point of access to search for Technology Appraisals conducted globally. The database is maintained by volunteers.	The CRD HTA database was initially funded by the NIHR in the United Kingdom, but it has not been updated since March 31, 2018. The database will remain accessible as an archive until at least March 2024.
HTA coverage	The INAHTA website claims that there are 120 HTA bodies with records listed in the database, but it does not provide a list of the countries/guidance‐producing agencies.	Database content was supplied by 52 members of INAHTA and 20 other international HTA organizations.
Database inclusion criteria	Completed and ongoing Health Technology Assessments published by INAHTA and non‐INAHTA members are eligible for inclusion. All new content is checked by a database content administrator. An inclusion criterion based on quality is not applied and reports are not critically appraised.	Any item designated as a Health Technology Assessment by the contributing organization was eligible for inclusion. Context was checked by Centre for Reviews and Dissemination staff.
Dates of coverage	Not reported	Not reported
Number of records	As of April 2020, the INAHTA database indexed 16,768 completed Technology Appraisals.	Not reported

Abbreviations: CRD, Centre for Reviews and Dissemination; HTA, Health Technology Assessment; INAHTA, International Network of Agencies for Health Technology Assessment; NIHR, National Institute for Health Research.

This exploratory study set out to test the single‐point‐of‐access claim by comparing the coverage of the INAHTA and CRD HTA databases with direct searching on the NICE website.

## 2 METHODS

### 2.1 Study data

Data were NICE Technology Appraisals, defined as guidance produced by NICE to appraise a product, device, or technology. [1]

### 2.2 Identifying data

A search of the NICE website (https://www.nice.org.uk/) was conducted on April 21, 2022, by navigating to guidance and limiting the search to “Technology appraisal guidance.” View was changed to ‘all’, and data (NICE Technology Appraisal records) were copied to the clipboard and downloaded to Microsoft Excel. A total of 601 Technology Appraisals were identified and downloaded.

### 2.3 Analysis

Data were randomized in Microsoft Excel. The first 20 Technology Appraisals were chosen for analysis, irrespective of the technology or date of publication. Limiting to 20 Technology Appraisals was based on the resources available for this study.

The 20 Technology Appraisals were then searched for in both the INAHTA and CRD HTA databases. Both databases were searched using the full title of the Technology Appraisal within the search field, with no limits applied and the “all” (INAHTA)/“any fields” (CRD) criterion selected (Chris Cooper, CC) or the “title” criterion (Sabrina Smith, SS). Where no Technology Appraisal was returned, a broader search focusing on the intervention of interest only was executed. The purpose of this second search was to check for any errors in the study record which might cause a title search to fail. The study accounted for the last update of the CRD HTA database on March 31, 2018, by removing Technology Appraisal records published after this date from the analysis of CRD HTA database coverage (N/A was used to indicate these records).

CC was located in London, England, and searched both databases via FireFox and again by Google Chrome using an Apple MacBook Pro (Monterey 12.3.1). [4] The searches were independently double‐checked by SS on May 17, 2022, June 10, 2022, and June 23, 2022. SS was located in Halifax, Nova Scotia, Canada, and searched the databases via Google Chrome using a Lenovo desktop PC (Windows 10 Pro, Version 21H2).

### 2.4 Interpretation

If one of the 20 NICE Technology Appraisals in the random sample was identified in the INAHTA or CRD HTA database, this was a positive finding, as it suggests comparable coverage of Technology Appraisals between the NICE website and the single‐point‐of‐access databases.

If a Technology Appraisal could not be identified in the INAHTA or CRD HTA databases, this was a negative finding, suggesting that the coverage of the INAHTA or CRD HTA databases is incomplete for our sample.

It was noted if a Technology Appraisal was identified in one database but not the other, as this information could be used to determine any differences between the coverage of the two databases (aside from the obsolescence of the CRD HTA database as noted earlier).

### 2.5 Findings

This is an exploratory study based on a small sample size. The sample Technology Appraisals were published between October 2002 and November 2021.

Coverage was incomplete in both databases (Table 2). In the INAHTA database, 15 of 20 Technology Appraisals (75%) were not identified via full‐title or intervention‐specific searches. In the CRD HTA database, 7 of 12 Technology Appraisals (58%) that were published before the last update of the database were not identified.

**Table 2:** TA coverage across INAHTA and CRD HTA databases.

#	Guidance	TA #	Published	Last update	Identified in		Notes	Detailed search results
					INAHTA database	CRD HTA database		
1	Clopidogrel and modified‐release dipyridamole for the prevention of occlusive vascular events [5]	TA210	15‐Dec‐10	15‐Dec‐10	✓	✓	—	—
2	Myocardial perfusion scintigraphy for the diagnosis and management of angina and myocardial infarction [6]	TA73	26‐Nov‐03	01‐Jul‐11	✓	✓	—	—
3	Routine antenatal anti‐D prophylaxis for women who are rhesus D negative [7]	TA156	27‐Aug‐08	27‐Aug‐08	✗	✗	—	INAHTA: Full title = 0 “Rhesus D negative” = 3; no TA; TA156 embedded in TA41 record “Anti‐D prophylaxis” = 7; no TA; TA156 embedded in TA41 record CRD: Full title = 0 “Rhesus D negative” = 0 “Anti‐D prophylaxis” = 11; no TA; TA156 embedded in TA41 record
4	Adefovir dipivoxil and peginterferon alfa‐2a for the treatment of chronic hepatitis B [8]	TA96	22‐Feb‐06	22‐Feb‐06	✓	✓	—	—
5	Guidance on the use of drugs for early thrombolysis in the treatment of acute myocardial infarction [9]	TA52	30‐Oct‐02	30‐Oct‐02	✓	✓	—	—
6	Rituximab for the first‐line maintenance treatment of follicular non‐Hodgkin's lymphoma [10]	TA226	22‐Jun‐11	22‐Jun‐11	‡	‡	NIHR reference to the NICE TA	INAHTA: Full title = 1; no TA; NIHR reference to the NICE TA but no indication of TA# Same result as above for: “Follicular non‐Hodgkin's lymphoma” = 13 “Rituximab AND Follicular non‐Hodgkin's lymphoma” = 8 “Rituximab AND lymphoma” = 28 CRD: Full title = 0 “Follicular non‐Hodgkin's lymphoma” = 1; no TA Rituximab AND Follicular non‐Hodgkin's lymphoma” = 0 “Rituximab AND lymphoma” = 23; no TA; NIHR reference to the NICE TA but links broken and no indication of TA#
7	Prasugrel with percutaneous coronary intervention for treating acute coronary syndromes [11]	TA317	23‐Jul‐14	23‐Jul‐14	✗	✗	NIHR reference to out‐of‐date NICE TA	INAHTA: Full title = 2; no TA; identified NIHR review of TA182 (which was replaced by TA317) but not TA317 “Prasugrel” = 11; TA182 identified but not TA317 CRD: Full title = 0 “Prasugrel” = 20; identified NIHR review of TA182 (which was replaced by TA317) but not TA317
8	Naloxegol for treating opioid‑induced constipation [12]	TA345	22‐Jul‐15	22‐Jul‐15	‡	‡	NIHR reference to the NICE TA	INAHTA: Full title = 1; No TA; identified NIHR reference to TA345 Same result as above for: “Naloxegol” = 2 CRD: Full title = 0 “Naloxegol” = 2; no TA; identified NIHR reference to TA345
9	Isatuximab with carfilzomib and dexamethasone for treating relapsed or refractory multiple myeloma (terminated appraisal) [13]	TA727	22‐Sept‐21	22‐Sept‐21	✗	N/A	Searched without (terminated appraisal) n = 0; searched for “Isatuximab with carfilzomib” n = ; and “Isatuximab” n = 0.	INAHTA: Full title, searched without (terminated appraisal) = 16; no relevant results “Isatuximab with carfilzomib” = 0 “Isatuximab”= 0 CRD: N/A
10	Crizanlizumab for preventing sickle cell crises in sickle cell disease [14]	TA743	03‐Nov‐21	03‐Nov‐21	✗	N/A	—	INAHTA: Full title = 0 “Crizanlizumab” = 1; no TA CRD: N/A
11	Ixekizumab for treating axial spondyloarthritis [15]	TA718	21‐Jul‐21	21‐Jul‐21	✗	N/A	—	INAHTA: Full title = 0 “Ixekizumab” = 3; no relevant results CRD: N/A
12	Rivaroxaban for the prevention of stroke and systemic embolism in people with atrial fibrillation [16]	TA256	23‐May‐12	02‐Jul‐21	‡	‡	NIHR reference to the NICE TA	INAHTA: Full title = 1; No TA; NIHR reference to the NICE TA report “Rivaroxaban” = 14; No TA; NIHR reference to the NICE TA report CRD: Full title = 1; No TA; NIHR reference to the NICE TA “Rivaroxaban” = 55; No TA; NIHR reference to NICE TA
13	Anakinra for treating Still's disease [17]	TA685	31‐Mar‐21	31‐Mar‐21	✗	N/A	—	INAHTA: Full title = 0 “Anakinra” = 6; no TA CRD: N/A
14	Durvalumab in combination for untreated extensive‐stage small‐cell lung cancer (terminated appraisal) [18]	TA662	25‐Nov‐20	25‐Nov‐20	✗	N/A	—	INAHTA: Full title = 0 “Durvalumab” = 1; no TA CRD: N/A
15	Treosulfan with fludarabine for malignant disease before allogeneic stem cell transplant [19]	TA640	05‐Aug‐20	05‐Aug‐20	✗	N/A	—	INAHTA: Full title = 0 “Treosulfan” = 0 CRD: N/A
16	Ertugliflozin with metformin and a dipeptidyl peptidase‐4 inhibitor for treating type 2 diabetes [20]	TA583	05‐Jun‐19	05‐Jun‐19	✗	N/A	—	INAHTA: Full title = 0 “Ertugliflozin” = 0 CRD: N/A
17	Dinutuximab beta for treating neuroblastoma [21]	TA538	22‐Aug‐18	22‐Aug‐18	✗	N/A	—	INAHTA: Full title = 0 “Dinutuximab” = 2; no TA CRD: Full title = 0 “Dinutuximab” = 1; no TA
18	Holoclar for treating limbal stem cell deficiency after eye burns [22]	TA467	16‐Aug‐17	16‐Aug‐17	✗	✗	—	INAHTA: Full title = 0 “Holoclar” = 1; no TA CRD: Full title = 0 “Holoclar” = 1; no TA
19	Percutaneous vertebroplasty and percutaneous balloon kyphoplasty for treating osteoporotic vertebral compression fractures [23]	TA279	24‐Apr‐13	24‐Apr‐13	‡	‡	NIHR reference to the NICE TA	INAHTA: Full title = 0 “Percutaneous vertebroplasty” = 25; no TA; identified reference to NIHR report CRD: Full title = 0 “Percutaneous vertebroplasty” = 40; no TA; identified reference to NIHR report
20	Alitretinoin for the treatment of severe chronic hand eczema [24]	TA177	26‐Aug‐09	26‐Aug‐09	✓	✓	—	—

*Note*: ✓, NICE TA guidance identified in database; ‡, Reference to NICE TA guidance identified in a non‐NICE record indexed in the database; and ✗, NICE TA guidance not identified. Data were correct on dates of search (see Methods).

Abbreviations: CRD, Centre for Reviews and Dissemination; HTA, Health Technology Assessment; INAHTA, International Network of Agencies for Health Technology Assessment; N/A, not applicable; NICE, National Institute for Health and Care Excellence; NIHR, National Institute for Health Research.

Both databases only returned the following five Technology Appraisals from the sample: TA210, TA73, TA96, TA52, TA177 (see Table 2). All of the retrieved Technology Appraisals were published before 2012. However, there were still gaps as there were two Technology Appraisals published before 2012 in the sample that were not identified in either database (i.e., five of seven Technology Appraisals published before 2012 were identified in both databases). This is in contrast to coverage after 2012—none of the sample Technology Appraisals published from 2012 onward were retrieved from the INAHTA database (13 of 20 in the sample) or the CRD HTA database (five of 12 in the sample).

Four of the Technology Appraisals that were not identified in the databases were referenced in alternate records, all of which were National Institute for Health and Care Research (NIHR) reports: TA226, TA345, TA256, and TA279 (see Table 2). These required the researcher to click a link that led to the TA on the NICE website. In the case of TA226 (Rituximab for the first‐line maintenance treatment of follicular non‐Hodgkin's lymphoma), the were no links to the NICE TA, only links that led to the NIHR Research Awards page. [25] On the CRD HTA database, the link was broken and the abstract record stated that the health technology appraisal was ongoing as of June 15, 2011. [26]

In the case of TA317 (Prasrugel with percutaneous coronary intervention for treating acute coronary syndromes), the TA was not identified but both databases identified an old NIHR record for a different Technology Appraisal—TA182. However, this Technology Appraisal was replaced by TA317 in 2014 and there was no indication in either database that the retrieved data were obsolete.

Coverage between the two single‐point‐of‐access databases was comparable overall, barring Technology Appraisals published after the last CRD HTA database update in 2018.

## 3 DISCUSSION

For the 20 Technology Appraisals that formed the study sample, we found that coverage is incomplete in both the INAHTA and CRD HTA databases. This raises concerns that neither database offers a reliable point of access to NICE Technology Appraisals.

The following discussion is focused on the INAHTA database, as the CRD HTA database is no longer being updated.

### 3.1 Implications for practice

The findings of this study suggest that the records for NICE Technology Appraisals in the INAHTA database need to be confirmed against the NICE website to ensure all NICE Technology Appraisals are available for retrieval in the INAHTA database in future updates. Further, some records that are returned via the INAHTA database are out of date, with no such indication, warranting a full search on the NICE website in any event. Ideally, this information would be added to records as updates are made to the database.

For researchers needing to identify NICE Technology Appraisals, a search of the NICE website is essential, and it should be performed as a priority over the INAHTA or CRD HTA databases.

### 3.2 Implications for research

INAHTA positions their database as a single point of access which reduces the complication and time needed to search the websites of individual guidance‐producing agencies. [3] This is a valuable service which is offered by volunteers; however, if searches do not yield comprehensive results, a researcher cannot rely on the database, making its utility questionable.

Extending our analysis to further Technology Appraisals from NICE, and examining the coverage of other agencies, would be a valuable next step. This would help further determine the coverage of the INAHTA database and indicate where updates should be made.

### 3.3 Limitations

This study only evaluated the coverage of the INAHTA and CRD HTA databases compared to the NICE website. This is not an evaluation of the coverage of other guidance‐producing agencies within those databases, although this is an important next step.

This evaluation focused on only 20 Technology Appraisals due to the resources available for the research. Although this is a limited sample of Technology Appraisals, the percentage of missed Technology Appraisals for both resources suggests a broader issue with the databases.

The CRD HTA database has not been updated since 31 March 2018. Due to the randomization of Technology Appraisals for analysis, seven of the Technology Appraisals were ineligible for comparison in the CRD HTA database because they were published after the database was last updated. The study accounted for this; however, it must be acknowledged that the sample size for this database was reduced as a result of the database being obsolete.

INAHTA is managed by volunteers. We acknowledge that resources that are free at point of use can be subject to funding and resource restrictions that might not be observed with commercial initiatives.

## 4 CONCLUSION

Findings indicate that researchers seeking to identify NICE Technology Appraisals should search the NICE website directly. How this finding impacts identification of guidance from other agencies should be evaluated.

## AUTHOR CONTRIBUTIONS

**Christopher Cooper**: Conceptualization; data curation; formal analysis; investigation; methodology; project administration; resources; validation; writing—original draft; writing—review and editing. **Sabrina Smith**: Data curation; formal analysis; investigation; methodology; project administration; resources; validation; visualization; writing—original draft; writing—review and editing.

## CONFLICT OF INTEREST STATEMENT

The authors declare no conflict of interest.

## PEER REVIEW

The peer review history for this article is available at https://www.webofscience.com/api/gateway/wos/peer-review/10.1002/cesm.12034.

## Data Availability

Data derived from public domain resources.
